# Efficient Sampling in Fragment-Based Protein Structure Prediction Using an Estimation of Distribution Algorithm

**DOI:** 10.1371/journal.pone.0068954

**Published:** 2013-07-25

**Authors:** David Simoncini, Kam Y. J. Zhang

**Affiliations:** Zhang Initiative Research Unit, Institute Laboratories, RIKEN, Wako, Saitama, Japan; University of Michigan, United States of America

## Abstract

Fragment assembly is a powerful method of protein structure prediction that builds protein models from a pool of candidate fragments taken from known structures. Stochastic sampling is subsequently used to refine the models. The structures are first represented as coarse-grained models and then as all-atom models for computational efficiency. Many models have to be generated independently due to the stochastic nature of the sampling methods used to search for the global minimum in a complex energy landscape. In this paper we present 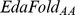, a fragment-based approach which shares information between the generated models and steers the search towards native-like regions. A distribution over fragments is estimated from a pool of low energy all-atom models. This iteratively-refined distribution is used to guide the selection of fragments during the building of models for subsequent rounds of structure prediction. The use of an estimation of distribution algorithm enabled 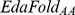 to reach lower energy levels and to generate a higher percentage of near-native models. 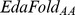 uses an all-atom energy function and produces models with atomic resolution. We observed an improvement in energy-driven blind selection of models on a benchmark of 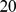 in comparison with the 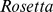 AbInitioRelax protocol.

## Introduction

The prediction of protein structures from their sequences has been a subject of intense research ever since the seminal work of Anfinsen [1]. Based on the principle that form follows function, the three-dimensional (3D) structure of a protein provides critical clues to its function. Moreover, the knowledge of a protein structure facilitates the design of therapeutic agents that modify its function. There are two main challenges that a protein structure prediction (PSP) method has to face: the inaccuracy in energy functions and the size of the search space. The inaccuracy in energy functions make identification of near-native models a difficult task. A study suggests that in some cases the native structure does not belong to the global minimum basin [2], and it was estimated that for over 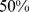 of the tested targets, a better sampling of the search space may lead to successful predictions. Inaccuracies in the energy functions combined with the huge size of the conformational space give rise to sampling issues. Many methods have been proposed to deal with these problems. One idea was to reduce the search space by assembling the structure from a pool of experimentally determined structural fragments [3]. This fragment assembly approach has become one of the most popular methods for protein structure prediction due to the success of 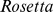 [4], [5], [6]. 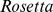 employs a two stage strategy: fragment assembly followed by all-atom refinement. During the fragment assembly, the protein models are represented by backbone atoms and centroid of side chains (coarse-grained sampling). Once the models are assembled, the structure representation is switched to all-atom: side chains are added and packed by minimizing an all-atom knowledge-based energy function [7].

Many other fragment-based approaches have been proposed. The 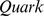 method, which was successful in recent CASP experiments [8], uses variable length fragments and replica exchange Monte Carlo for sampling [9]. 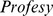 attempts to improve the conformational sampling efficiency by using Conformational Space Annealing [10]. 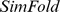 introduces the concept of reversible fragment insertion, where local structures created by the junction of two proteins fragments can be reused later on during the sampling process [11]. 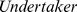 combines variable length fragments and fold recognition analysis. It uses a genetic algorithm for sampling [12]. 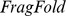 combines supersecondary structural fragments built from several sequential secondary structures with small fragments. The models are assembled using a genetic algorithm and simulated annealing [13]. Some probabilistic methods estimate the joint angle distribution by a mixture model of particular distributions [14], [15]. Among them, *Fragment-HMM* uses protein fragments to obtain a first estimation of the torsion angle distributions using the cosine model, a bi-variate von Mises distribution. The cosine models are used as hidden nodes in a position-specific hidden Markov Model which is then used to sample a sequence of torsion angle pairs. The method then uses the generated protein models as new input to refine the joint angle distributions and iterates until convergence. The incorporation of information from prior rounds has been shown to be beneficial for the prediction of protein secondary and tertiary structures [16], [17]. Using the principle of sequential stabilization, Adhikari *et al.* has demonstrated that the accuracy of predicted structures can be greatly improved using a process of progressive learning and structural stabilization found in prior round of folding.

Fragment-based methods such as 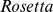 need to generate a huge number of models in order to find a correct structure. The rugged nature of the energy landscape, due to the inaccuracy of the energy functions and the size of the search space, necessitates the use of stochastic sampling methods. Recently, we have proposed a method (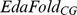) that takes advantage of the large size of the data-set by enabling communications between predictions during the sampling [18]. The idea was that if some fragments occur more often in low energy models, then these fragments are more likely to resemble the native structure. Using an Estimation of Distribution Algorithm (EDA), 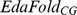 iteratively updates the probabilities of inserting fragments in new models according to their frequency in low energy models. The results obtained with 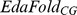 were promising, and show that the method is able to enhance the proportion of near-native structures in the pool of protein models on a benchmark of 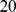 proteins. However, it employed coarse-grained models to estimate fragment probabilities. As a result, some high quality models could not be identified and the sampling was misguided on a few targets because of energy function inaccuracy. Also, as its aim was to study the sampling dynamics at a coarse-grained level, it lacks the ability to produce all-atom models.

In this paper, we describe a new method (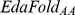) that estimates probability mass functions of fragments from all-atom models instead of coarse-grained models as 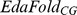. Our new protocol relies on 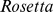
's all-atom energy in order to rank the models during the estimation of distribution step of the algorithm and the all-atom models produced show notable improvements over 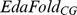 and 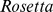 AbInitioRelax. The gain in accuracy provided by the all-atom energy function had several positive effects on the sampling; the closest structure to native in the pool of models, the proportion of near-native models and the accuracy of lowest energy models improved on average, and on a majority of the 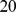 proteins in our benchmark.

## Results


**Figure 1:**
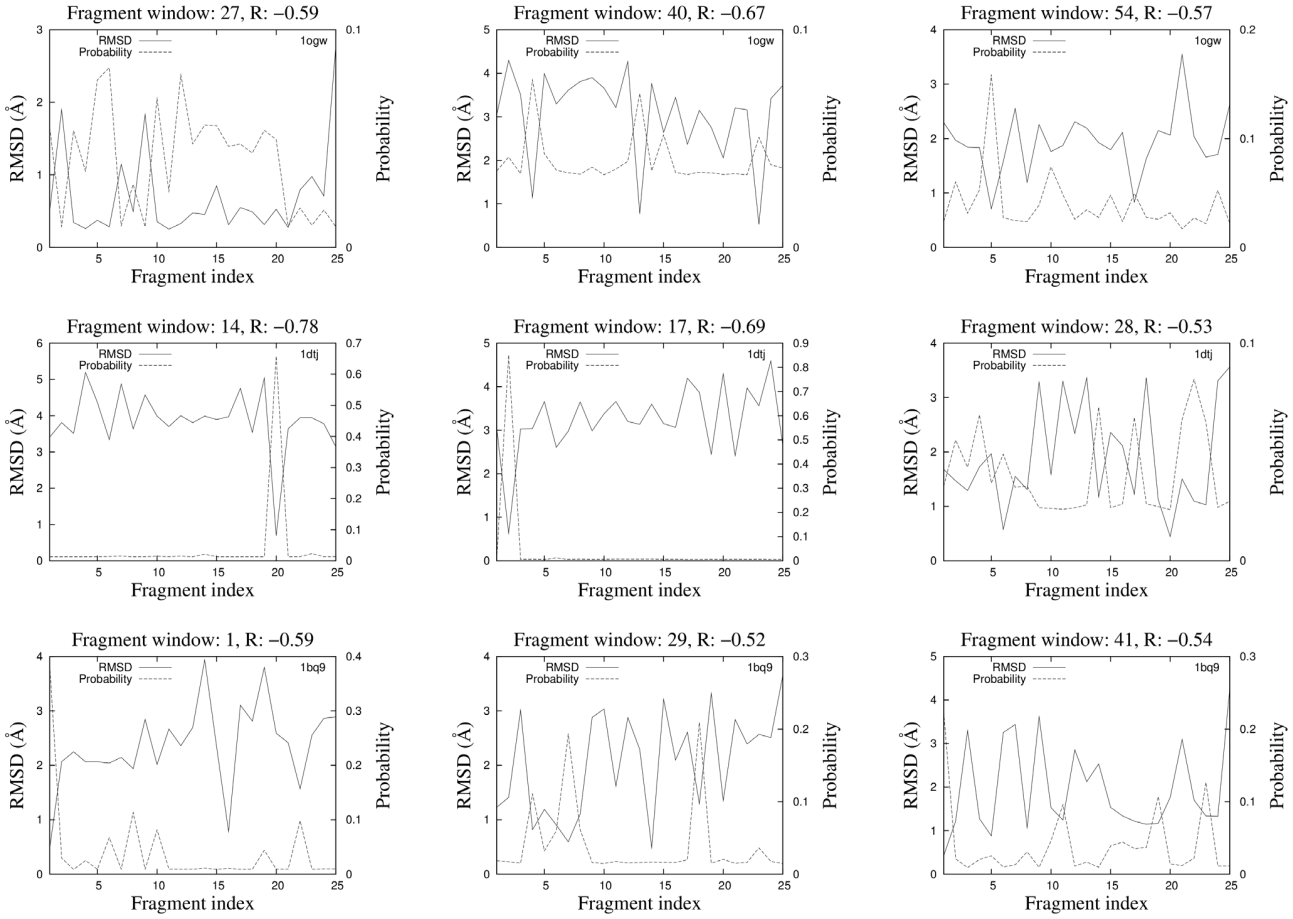
Estimation of distribution: the probability of selecting each of the 25 9-residue fragments at iteration 4 is plotted against the C*_α_*RMSD of each fragment to the native structure for 3 fragment windows of PDB codes 1ogw, 1dtj and 1bq9. The Pearson correlation coefficient (R) between probabilities and C*_α_*RMSD to native structure is given for each fragment window.

**Figure 2:**
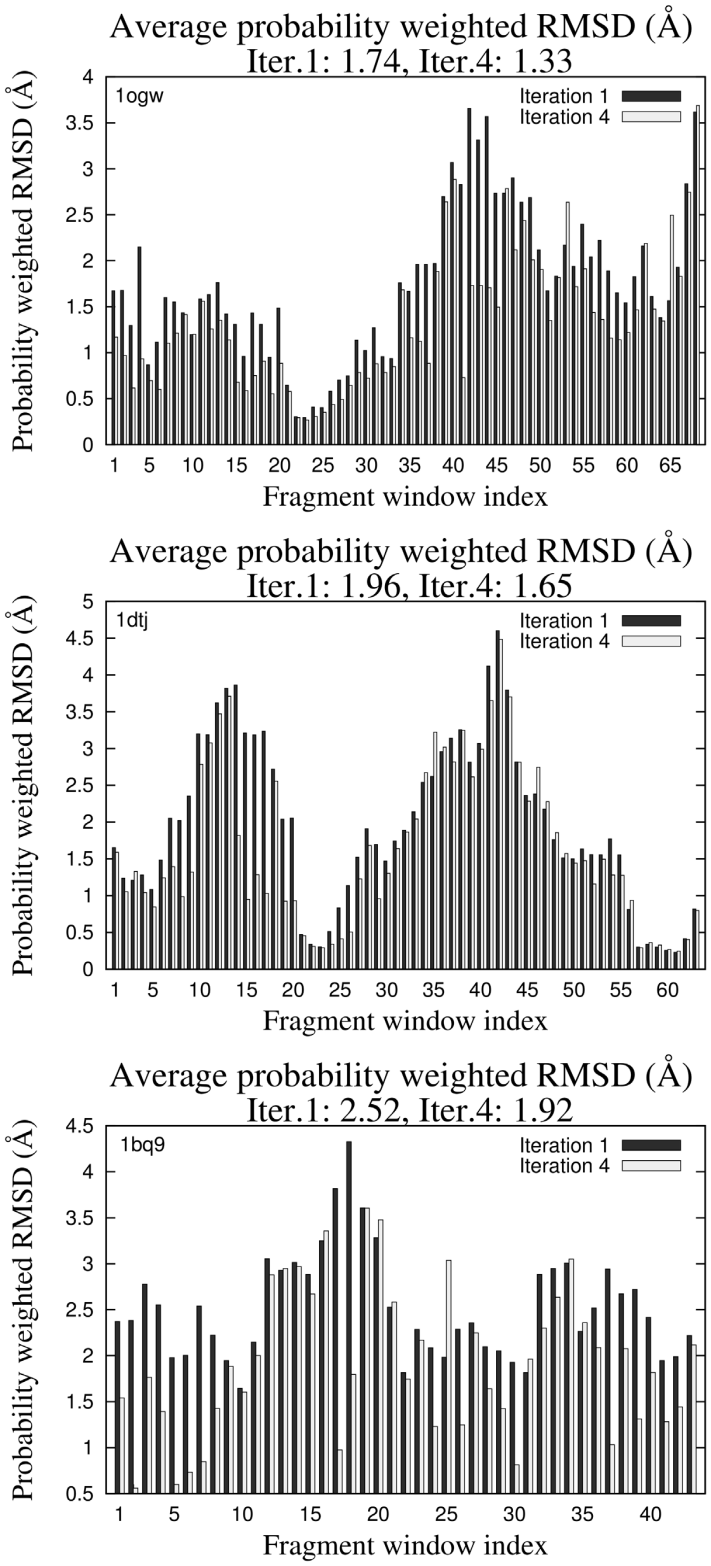
Average probability weighted C*_α_*RMSD : for each fragment window, the average of the C*_α_*RMSD of each fragment weighted by the probability of selecting it is plotted for iterations 1 and 4. The average over all fragments windows at iterations 1 and 4 shows an overall improvement on all cases: PDB codes 1ogw, 1dtj and 1bq9.


**Figure 3:**
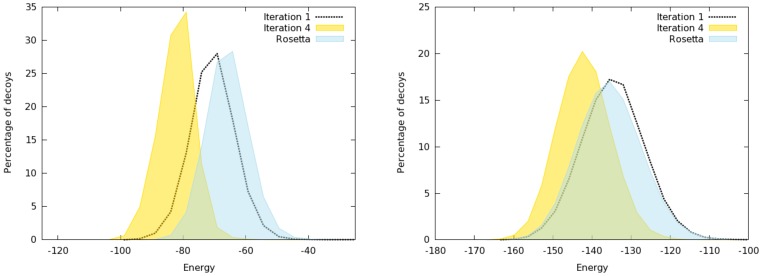
Histograms of energy distribution: comparison between iterations 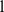, 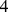 of 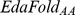 and 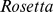 for 1bq9 (left) and 1ogw (right). The estimation of distribution algorithm allows 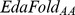 to increase the performance from iteration 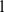 to iteration 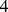.

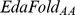
 favors fragments from the library that are closer to native fragments. The probability of selecting each of the 25 9-residue fragments at iteration 4 of 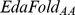 is plotted against the C*_α_*RMSD of each fragment to the native structure for 3 fragment windows of PDB codes 1ogw, 1dtj and 1bq9 in Figure 1. Pearson product moment correlation coefficient shows anti-correlation between probabilities and C*_α_*RMSD to native, putting in evidence that native-like fragments usually get a high probability of being selected and *vice versa*. The average C*_α_*RMSD of fragments to native weighted by their probability of being selected at iterations 1 and 4 is plotted for the same PDB codes in Figure 2. Overall, the average probability weighted C*_α_*RMSD shows improvement from iteration 1 to 4 in all of the 3 cases.

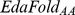
 can successively generate lower energy models after each iteration. The distributions of the energies of the models generated by 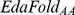 at iterations 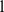 and 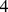 and by 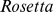 were shown in Figure 3. The measures were made with two protein targets, PDB codes 1bq9 and 1ogw. Whereas the distributions of 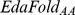
's iteration 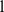 and 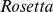 have the same shape and suggest that the two methods sample similar regions of the search space and in equal proportions, the curve describing the iteration 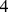 is different. In both cases, 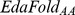 favors the sampling of low energy basins of the search space. For 1bq9, even though the shape of the curves are the same, we note that the curve at iteration 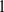 from 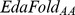 already shifts a little towards lower energies.

**Figure 4:**
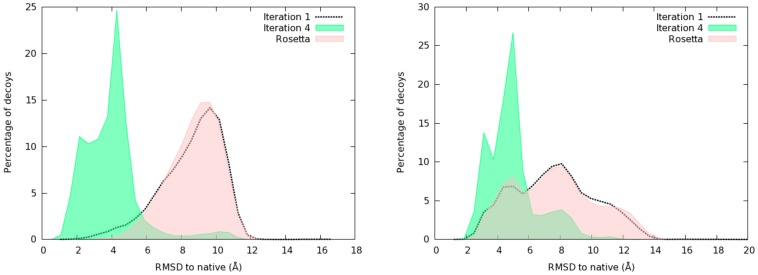
Histograms of C*_α_*RMSD to native distribution: comparison between iterations 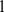, 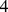 of 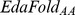 and 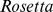 for 1bq9 (left) and 1ogw (right). Whereas the distributions look identical between iteration 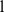 and 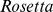, at iteration 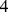 the distribution has shifted towards native structure.

The improvement in lower energy basins sampling achieved by 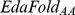 translates into better quality models with lower C*_α_*RMSD (C-alpha Root Mean Square Deviation) to native. This can be seen from Figure 4, which plots the distribution of decoys as a function of C*_α_*RMSD to native. The results match with the observations made at energy level. At first iteration, 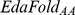 sample similar regions of the search space. As a shift at iteration 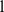 for 1bq9 was observed, it can be seen in this figure that 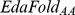 produces models closer to the native structure even at iteration 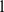. The distributions change at iteration 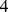. In both cases, regions closer to the native structure are thoroughly sampled at this stage. The algorithm is particularly efficient for 1bq9. This efficiency of the algorithm could be due to the shape of the fitness landscape induced by 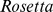 all-atom energy. Since 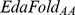 favors the insertion of fragments which have been identified as being helpful in minimizing the energy, a good correlation between the lowest energies and C*_α_*RMSD to native is one reason for improved performance.

**Figure 5:**
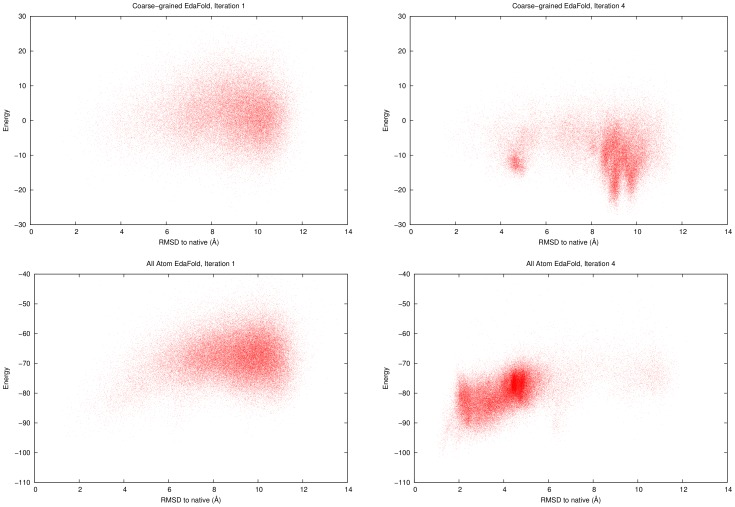
Fitness landscapes at iterations 1 and 4 with coarse-grained models (top, 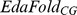) and all-atom models (bottom, 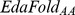) for 1bq9.

The quality of models have been improved by estimating probability mass functions of fragments based on all-atom energy instead of coarse-grained energy. The distribution of energies as a function of C*_α_*RMSD to native for all models generated at iteration 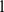 and 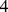 produced by 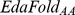 for one target, 1bq9, are shown in Figure 5. In addition, it also shows the scatter plot between energy and C*_α_*RMSD to native for the same target with 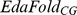 which was using 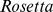
's coarse-grained energy. This figure revealed the critical importance of the energy function in our process. In 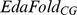, even though some good models were discovered, the sampling process was misled by the inaccuracies of the coarse-grained energy function. As a result, 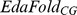 enhanced the search in the wrong region at about 9 Å from the native structure. The high number of low energy misfolded models complicates the identification of the high quality ones. Once we use the all-atom energy in our process, the fitness landscape changes. This time, the algorithm can discover unprecedented energy levels which correspond to models located at about 1 Å C*_α_*RMSD from the native structure. This suggests that using a more accurate energy function for the periodic estimation of distributions in 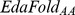 improves the sampling.

We know that turning coarse-grained models into all-atom models dramatically modifies the landscape, but it is unclear if using the all-atom energy to drive 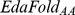
's sampling is more efficient. We performed some control experiments for which we used 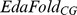 to generate coarse-grained models, and turned them into all-atom models with 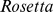
's fast Relax protocol as a final step. The results were then compared with those from our new 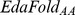 algorithm which includes fast Relax at each iterative step. The same number of models was produced with each method for 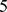 protein sequences and each protocol's ability to produce near-native models was examined. Table 1 shows that 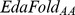 systematically produced models with smaller C*_α_*RMSD to the native structure, and 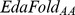 also generated a higher proportion of near-native models than 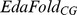. This result shows that switching the models to an all-atom representation at each iteration of our algorithm and modifying the probabilities of subsequent fragment selection according to 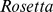
's all-atom energy function improves the accuracy of 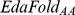.

**Table 1:** Comparison of all-atom models generated using fragment distributions estimated from all-atom models versus coarse-grained models.

	1‰ C*_α_*RMSD (Å)		1% C*_α_*RMSD (Å)		Best model (Å)	
Target	*EdaFold_AA_*	*EdaFold_CG_*	*EdaFold_AA_*	*EdaFold_CG_*	*EdaFold_AA_*	*EdaFold_CG_*
1di2	**0.70**	0.75	**0.86**	0.99	0.51	0.54
1scj	3.26	**3.14**	4.08	**3.44**	2.35	2.41
1tig	**3.28**	3.33	3.74	3.74	1.75	2.17
4ubp	**4.13**	4.27	**4.90**	5.16	3.03	3.04
1acf	**3.20**	3.67	**3.94**	4.32	2.20	2.44

1‰ C*_α_*RMSD is the average over the 1‰ lowest C*_α_*RMSD to native models. Similarly, 1% C*_α_*RMSD is the average over the 1% lowest C*_α_*RMSD to native models. Best model is the single lowest C*_α_*RMSD to native model. 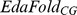 is a dataset of models obtained by generating coarse-grained 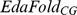 followed by 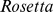
's fast Relax protocol. In 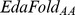, the fast Relax protocol is embedded in each iteration and contributes to the estimation of distributions. Data in bold are statistically better with a confidence greater than 95% according to the Student's *t*-test.


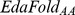
 can generate a higher proportion of near-native models on average. The Table 2 shows a comparison of the C*_α_*RMSD to native that each method can reach. The average lowest 1 C*_α_*RMSD and 1% C*_α_*RMSD to native are shown. In addition, the C*_α_*RMSD to native of the best model is shown. The analysis of the proportion and quality of near-native models produced by 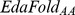 and 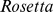 shows that 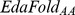 can generate a higher proportion of near-native models on average. The best model generated by each method without selection considerations is also slightly better on average for 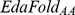. Table S1 shows that the trend is similar when looking at AARMSD (All-Atom Root Mean Square Deviation) values. The trend is the same whether we compare C*_α_*RMSD or AARMSD.

**Table 2:** Comparison of all-atom models generated by 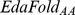 and 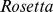.

	1‰ C*_α_*RMSD (Å)		1% C*_α_*RMSD (Å)		Best model (Å)	
Target	*EdaFold_AA_*	Rosetta	*EdaFold_AA_*	Rosetta	*EdaFold_AA_*	Rosetta
1bq9	**1.29**	3.33	**1.75**	4.51	0.98	2.27
1di2	**0.70**	0.91	**0.86**	1.43	0.51	0.59
1scj	**3.26**	3.53	**4.08**	4.22	2.35	2.42
1hz5	2.26	**2.21**	2.52	**2.46**	1.24	1.78
1cc8	**2.09**	2.46	**2.40**	3.13	1.72	1.77
1ctf	3.44	**3.09**	4.36	**3.76**	2.70	2.40
1ig5	**2.24**	2.29	**2.61**	2.68	1.69	1.74
1dtj	2.46	**2.41**	3.56	**3.47**	1.35	1.47
1ogw	**2.25**	2.67	**2.72**	3.10	1.33	1.79
1dcj	**2.55**	2.68	**3.02**	3.38	2.00	1.65
2ci2	3.18	**2.95**	4.81	**4.15**	2.24	2.07
3nzl	**3.63**	3.83	**4.11**	4.45	3.07	2.96
1a19	**2.90**	3.28	**3.55**	4.34	2.20	1.99
1tig	3.28	**3.20**	**3.74**	3.89	1.75	2.31
1bm8	3.76	**3.58**	4.79	**4.55**	2.84	2.43
4ubp	4.13	**3.86**	4.90	**4.70**	3.03	2.48
1m6t	**1.22**	1.46	**1.42**	1.84	1.01	1.08
1iib	**2.92**	3.30	**4.00**	4.82	1.85	1.89
1acf	**3.20**	4.55	**3.94**	5.85	2.20	2.77
3chy	**3.51**	3.82	**4.63**	4.93	2.18	2.52
Average	**2.72**	2.97	**3.39**	3.79	1.91	2.02

1‰ C*_α_*RMSD is the average over the 1‰ lowest C*_α_*RMSD to native models. Similarly, 1% C*_α_*RMSD is the average over the 1% lowest C*_α_*RMSD to native models. Best model is the single lowest C*_α_*RMSD to native model. Data in bold are statistically better with a confidence greater than 95% according to the Student's *t*-test.

**Figure 6:**
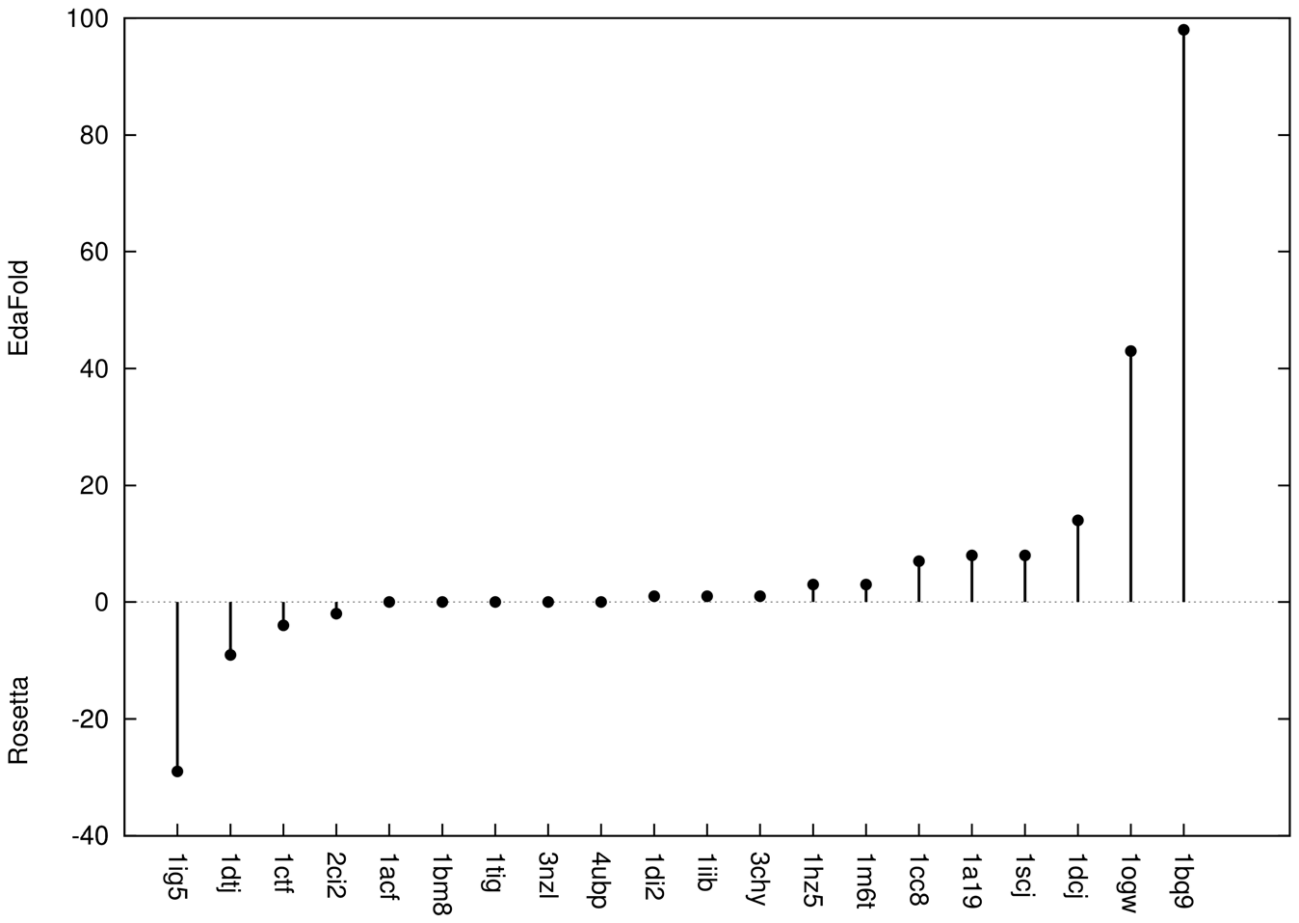
Percentage of near native models: 100 lowest energy models were selected from 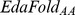
's and 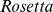
's datasets. The percentage of models less than 3 Å away from native in terms of C*_α_*RMSD was computed. The differences in percentages between 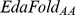 and 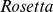 are plotted.

The improvement of low quality models away from native is probably not very meaningful. Models with C*_α_*RMSD less than 3 Å from native structure could potentially be used as templates for solving crystal structures by molecular replacement [19]. A global view of the ability of each method to produce and identify near-native models was given in Figure 6. The percentage of models within 3 Å C*_α_*RMSD of the native structure for the 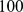 lowest energies were computed. For each target, the difference between the percentage of near-native models obtained from 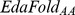
's and 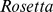
's dataset was plotted. This difference is in favor of 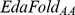 for all points above the 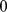 straight line. It is in favor of 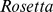 for all points under it. A majority of the points are above the straight line, which confirms that 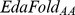 improves the quality of the lowest energy models. The percentage of models generated closer than 3 Å C*_α_*RMSD from the native structure amongst the 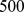 lowest energies in 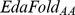
's and 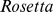
's datasets was analyzed in details and illustrated in Figure S1. Measurements range from models within 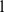 Å to less than 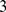 Å from native by steps of 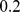 Å. 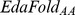 was able to produce a higher percentage of models at less than 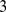 Å for 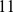 targets. 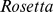 outperforms 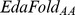 on 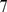 targets. Neither of the two methods was able to generate near-native models within the 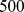 lowest energies on the same two targets: PDB codes 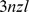 and 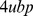.

The improved distribution of low energy models has enabled the “blind selection” of better models amongst all generated by 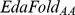. The “blind selection” results with energy as a selection criterion for 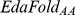 and 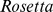 are shown in Table 3. Two results, the first and best predictions, are presented. The first prediction is the model with the lowest energy in the dataset whereas the best prediction is the best model out of the 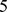 lowest energies. Both first and best prediction of 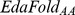 improves over 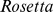 of about 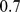 Å either when looking at C*_α_*RMSD or AARMSD (See Table S2 for AARMSD values).

**Table 3:** Comparison of best all-atom models selected based on energy.

	First prediction (Å)		Best prediction (Å)	
Target	*EdaFold_AA_*	Rosetta	*EdaFold_AA_*	Rosetta
1bq9	1.55	4.32	1.38	4.32
1di2	1.00	1.23	0.76	0.86
1scj	7.74	7.23	3.61	6.36
1hz5	3.21	3.51	3.00	3.18
1cc8	3.89	8.28	3.66	3.29
1ctf	7.05	4.84	4.58	2.76
1ig5	6.46	2.64	3.63	2.64
1dtj	1.72	1.72	1.69	1.72
1ogw	2.47	2.71	2.47	2.71
1dcj	5.02	3.02	2.50	2.56
2ci2	7.73	8.47	6.77	6.41
3nzl	5.95	5.80	5.95	5.33
1a19	2.73	3.76	2.73	3.10
1tig	4.07	3.92	3.69	3.72
1bm8	9.03	3.73	3.44	3.73
4ubp	10.48	10.50	5.87	8.51
1m6t	1.99	1.94	1.34	1.88
1iib	2.50	15.28	2.50	9.46
1acf	3.60	2.77	3.00	2.77
3chy	4.38	12.37	4.38	5.38
Average	4.63	5.40	3.35	4.04

The first prediction is the model with the lowest energy. The best prediction is the best model out of the five lowest energies. The C*_α_*RMSD to native structure for predicted models are shown.

Finally, the improved “blind selection” of models is due to the iterative estimation of distribution over the fragments. A comparison of the blind selection ability of 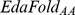 at iterations 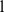 and 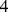 is given in Table 4. The first and best of 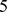 predictions are shown for models taken from iteration 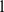 alone and iteration 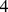 alone. As stated in the method section, 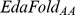 produces one quarter of the total number of models at each iteration. The results show an improvement of 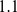 Å on average for the first prediction and over 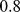 Å on average for the best prediction. For comparison, one quarter of the final models generated by 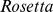 AbInitioRelax was randomly selected. The results show that 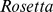 performs slightly better than 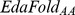 at iteration 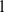, when no information is shared to produce the models. This result suggests that the sampling algorithm of 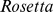 is more efficient than ours. The same trend was observed when looking at AARMSD values (see Table S3). Nevertheless, 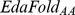 outperforms 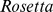 on this benchmark thanks to the use of EDA. Fragment-based approaches appear to gain considerable advantage from sharing information between predictions.

**Table 4:** Blind selection ability of all-atom models generated by 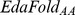 at iterations 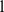 and 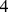.

	First prediction (Å)			Best prediction (Å)		
Target	Iter. 1	Iter. 4	Rosetta	Iter. 1	Iter. 4	Rosetta
1bq9	6.28	1.38	9.41	2.86	1.11	6.20
1di2	1.37	0.85	1.05	0.92	0.76	0.94
1scj	3.61	7.87	7.23	3.61	2.64	6.36
1hz5	3.39	3.24	3.98	2.98	3.15	3.21
1cc8	3.86	3.49	8.28	3.26	3.03	2.80
1ctf	5.81	7.05	4.84	4.78	5.62	3.39
1ig5	3.11	6.46	2.95	2.62	6.35	2.71
1dtj	3.22	1.68	1.72	1.69	1.65	1.72
1ogw	3.29	2.78	2.71	3.08	2.68	2.71
1dcj	2.85	5.02	3.90	2.85	2.68	2.68
2ci2	7.49	7.73	7.20	7.14	6.77	7.20
3nzl	10.87	11.42	5.80	5.58	5.50	4.99
1a19	6.77	3.82	3.76	3.26	2.75	3.10
1tig	4.60	3.69	4.29	3.91	3.69	4.29
1bm8	9.13	9.03	4.06	9.13	3.44	3.17
4ubp	9.23	11.39	10.50	5.93	7.64	7.74
1m6t	2.21	1.93	1.88	1.57	1.34	1.36
1iib	9.87	2.50	15.28	9.79	2.50	6.98
1acf	10.43	3.60	8.59	4.25	3.00	5.42
3chy	13.96	4.38	9.08	7.89	4.38	5.84
Average	6.07	4.97	5.83	4.35	3.54	4.15

The first prediction is the model with the lowest energy. The best prediction is the best model out of the five lowest energies. All results are shown as C*_α_*RMSD to native structure. Models produced at iteration 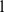 alone and iteration 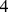 alone are compared. For comparison, the columns Rosetta show the same data obtained from a sample of Rosetta models randomly picked from Rosetta's prediction results.

## Discussion

The identification of near-native models is challenging due to the inaccuracy of the energy function. In our previous communication, we presented 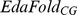 which focused on enrichment of the near-native structures at a coarse-grained level. We have demonstrated that the improved coarse-grained models can lead to better all-atom models by refining the top quality coarse-grained models into all-atom models [18]. However, the question of how top quality coarse-grained models are identified was not addressed. We were focused on the generation rather than the identification of good models, believing that it is pointless trying to identify good models if they are not generated to begin with. Here we show that it is not necessary to identify good coarse-grained models for refinement into all-atom models. Our new method takes all of the coarse-grained models and refines them into all-atom models. By interlacing all-atom representation of models and coarse-grained sampling, 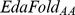 is able to enrich the proportion of near-native structures in the lowest energy all-atom models. Therefore, we have demonstrated here that the improved coarse-grained models can lead to better all-atom models without the need to identify good coarse-grained models.


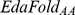
 uses the lowest energy all-atom models for the estimation of fragment distributions, whereas 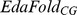 uses the lowest energy coarse-grained models for the estimation of fragment distributions. The estimated fragment distributions are used for the assembly of coarse-grained models in both methods. It is generally considered that the all-atom energy function is more accurate than the coarse-grained energy function. The all-atom models should be better than coarse-grained models given that the former has the benefit of complete side-chains taken into consideration. Therefore, it makes sense to derive probability mass functions of fragments from the refined all-atom models, and then to use these probabilities to guide the assembly of fragments. We have found that quality of coarse-grained models generated by 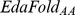 has been improved over those generated by 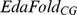 for the same target. By using this strategy, we benefit from the speed of the coarse-grained energy function during the sampling and of the accuracy of the all-atom energy which is helpful to guide the search in subsequent coarse-grained sampling rounds. Our results show the efficiency of this protocol for blind selection of models: at iteration 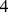 the quality of the lowest energy model and of the closest structure to native out of the top 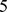 lowest energy models dramatically improves over iteration 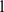.

The fragment assembly approach requires the generation of a huge number of models in order to produce a satisfactory solution. This is due to the stochastic nature of the sampling methods and to the inaccuracy of knowledge-based energy functions. The predictions are typically independent and modern technology allows massive parallelization of the computation. In this paper, we show that sharing information between these independent predictions can improve the quality of final results. The estimation of distribution over the fragment library relying on each fragment's frequency in low energy models allows 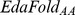 to significantly improve its performance in 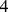 iterations. The amount of communications remains small, and the effective parallelization ratio is around 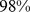. Our study suggests that the gain in performances is independent of the sampling method. Therefore, estimation of distribution can possibly increase the efficiency of any fragment-based approach.

The computation time required for the implementation of the estimation of distribution algorithm, including the encoding and decoding of fragments, the calculation of the probability mass functions and the sampling of fragments with Roulette wheels, is a small fraction of the time used for the generation of each model. In a previous communication, we reported that 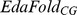 was 2.5 times slower than 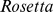. This was derived from the comparison of the times it took for the generation of equal numbers of coarse-grained models by both methods. The difference was due to our implementation of the simulated annealing and iterated hill climbing protocols in the coarse-grained model generation step is less efficient than the Monte Carlo search protocol in Rosetta. However, the extra time that 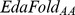 spent on the coarse-grained model generation did not produce better results than 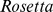 when EDA was not used as shown in Table 4. The quality of all-atom models from “iteration 1” without EDA was comparable or slightly worse than that of 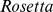 for both first prediction and best prediction. It is only when EDA was used that the all-atom models were improved as shown from “iteration 4” in Table 4 compared to 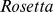 as well as “iteration 1”. Since our goal is to evaluate the impact of EDA on the all-atom models due to the improved coarse-grained model sampling and the computing time required for the implementation of EDA is negligibly small, the same number of all-atoms models were generated when comparing the performance of 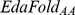 and 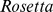 in this paper.

The incorporation of information from prior rounds in an iterative process has been shown previously to be a powerful technique applicable to protein structure prediction. The principle of sequential stabilization applied to protein folding is such an example [17]. The 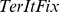 method uses the statistics of folding trajectories garnered from prior rounds to bias subsequent sampling of backbone dihedral angles, tertiary contacts and hydrogen bonds. There were no fragments used in the 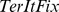 method.

Even though *Fragment-HMM* and 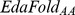 both use iterative strategies and are similar in the sense that they use information on generated models to refine estimations, the two methods differ in many ways. First, whereas *Fragment-HMM* estimates a distribution over the torsion angles assuming it follows a bi-variate von Mises distribution, 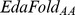 estimates a distribution over the fragment library starting from a uniform distribution. Then, the sampling method is different: *Fragment-HMM* uses a position specific hidden Markov Model and 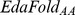 uses simulated annealing and iterated hill climbing. Also, even though *Fragment-HMM* initially uses fragments to estimate distributions at the first iteration, it doesn't use fragments during the sampling, unlike 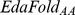. Finally, *Fragment-HMM* iterates until converging on one final model, whereas 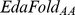 generates a diverse set of models, out of which the best ones will be selected.

The competition between global and local interactions plays a critical role in protein folding as well as structure prediction. This has been exploited for improving protein secondary and tertiary structure predictions using the principle of sequential stabilization [16], [17]. The identification of good quality fragments using the estimation of distribution algorithms implemented in 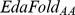 considers global interactions since the distributions are derived from the lowest energy all-atom models, whereas the initial fragment library is obtained by sequence homology that only takes into account local interactions of the residues within the fragment window. As the quality of all-atom models improve after each iteration, the global interactions are more accurately represented, which enables the good quality fragments being identified. However, unlike 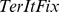 which can identify novel interactions as they have been generated and bias sampling towards those novel interactions, 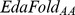 seeks only to bias existing fragments in the library, although novel fragments have been generated in the structure prediction process. The identification of novel fragments and bias the sampling towards good quality novel fragments will be an interesting direction for future exploration.

Our study also showed some deficiencies in 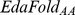
's sampling method. It fails to perform as well as 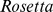 when no information is shared between the models (i.e. at iteration 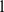). This observation leaves room for improvement of our method. Beyond the improvement of our heuristic sampling methods, future work will focus on other ways of sharing information between predictions.

## Methods


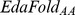
 is a fragment-based protein structure prediction algorithm. Similarly to 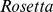 [7], it has two stages. First, 9-mers (followed by 3-mers) are assembled together to create coarse-grained models. 9-mers and 3-mers are taken from a fragment library which is created from protein structures available in the PDB. The fragment library we used was constructed using 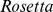
's fragment picking method [20]. When creating this library, proteins that shared more than 30% sequence identity with the target sequence were removed in order to remove any favorable bias. During the second stage, models are represented in atomic detail, and side chains are packed to minimize an all-atom energy function. The 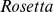 Relax protocol was used to perform this operation.


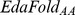
's protocol is described in Table 5 (Algorithm 1). It is an iterative algorithm using the concept of EDA to gather information between initially independent predictions. The concept of EDA is used to influence the probability of selecting fragments from the library. A fraction of the final model set (25%) is generated at each iteration. At iteration 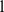, there is a uniform distribution over the library and every fragment has the same probability of being selected. At each iteration, a fraction of the lowest energy models (10%) is selected as a sample set. To compute the energy, all the models are relaxed in their all-atom representation via 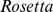 Relax protocol. We keep track of the links between coarse-grained and all-atom representations of a model so that we can retrieve which fragments were used to generate which all-atom model. The probabilities of selecting fragments for insertion during subsequent iterations are modified according to the observed distribution of fragments used in the sample set. The sampling is influenced by the probability mass function defined over the fragment library. Still, each iteration starts with models in extended conformation. Models generated at a given iteration are stored in the final set and are not reused for subsequent iterations. 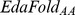 inherits its sampling engine from 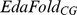 [18]. The sampling is performed using an alternation of simulated annealing [21] and iterated hill climbing [22]. The estimation of distribution is handled by the function 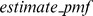 and is computed by the following formula:
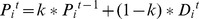
where 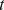 is the current iteration, 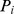 the probability of fragment 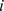, 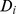 the observed frequency of fragment 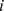 and 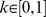 a conservation rate (k = 0.6 in our experiments).

**Table 5:** Algorithm 1: 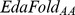 (comments are enclosed between braces).

**input** : *s* {sequence of the target protein}
**input** : *n* {number of minimization steps}
**output** : *p* {set of potential solutions}
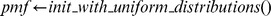
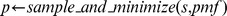 {first iteration}
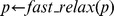
**for** *i* in 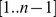 **do**
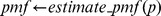
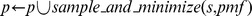 {remaining iterations}
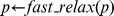
**end for**
**return** *p*

The performance of 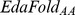 was measured on a dataset of 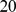 protein sequences, which were used in our previous studies [18]. We performed 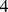 iterations of 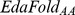 for each target. The models used during the estimation phase (iterations 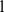, 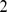 and 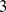) are part of the final model set: 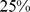 of the final models are generated at each iteration. The number of models generated (same for Rosetta and 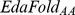) depends on the length of the target sequence: 250,000 for PDB codes 1m6t, 1iib, 1acf, 3chy and 300,000 for all other targets. C*_α_*RMSD calculations were performed with the *ranker* tool from Durandal [23]. All-atom RMSD, referred to as AARMSD in the following, were computed with the LSQKAB program from the CCP4 Software Suite [24], [25]. 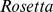 Version 3.2 was used for performance comparisons.

## Conclusions

We present an estimation of distribution-based protein structure prediction algorithm which generates models with atomic details. The use of 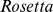
's fast Relax protocol in the iterative process of 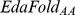 allows the estimation of distributions over the protein fragment libraries according to an all-atom energy function. Energy distribution and C*_α_*RMSD to native histograms revealed that our protocol can reach lower energies and generate more accurate models after 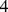 iterations. A comparison with 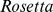 AbInitioRelax shows that 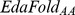 is able to produce more accurate models and a higher percentage of near-native structures. The proportion of near-native structures in the low energy range also improves on a majority of targets. As a result, energy-driven blind selection of models is more efficient: 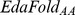 selects more accurate models when looking at the lowest or the top 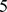 lowest energies in our dataset on a benchmark of 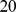 protein targets.

## Authors' Information


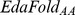
 is released under the GNU General Public License. It can be downloaded from http://www.riken.jp/zhangiru/software.html.

## Supporting Information

Figure S1
**Models distribution as a function of C** ***_α_*** **RMSD to native for the lowest 500 energies in** 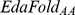 **and** 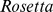 **datasets.**
(TIF)

Table S1
**Comparison of all-atom models generated by** 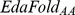 **and** 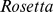 **.** 1‰ AARMSD is the average over the 1‰ lowest AARMSD to native models. Similarly, 1% AARMSD is the average over the 1% lowest AARMSD to native models. Best model is the single lowest AARMSD to native model. All mean differences are statistically significant with a confidence greater than 95% according to the Student's *t*-test.(PDF)

Table S2 **Comparison of best all-atom models selected based on energy.** The first prediction is the model with the lowest energy. The best prediction is the best model out of the five lowest energies. All results are shown as AARMSD to native structure.
(PDF)

Table S3
**Blind selection ability of all-atom models generated by** 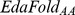 **at iterations** 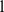 **and** 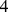 **.** The first prediction is the model with the lowest energy. The best prediction is the best model out of the five lowest energies. All results are shown as AARMSD to native structure. Models produced at iteration 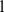 alone and iteration 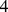 alone are compared. For comparison, the columns 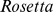 show the same data obtained from a sample of 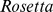 models randomly picked from 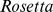
's prediction results.(PDF)

## References

[1] Anfinsen CB, Haber E, Sela M, White FH (1961) The kinetics of formation of native ribonuclease during oxidation of the reduced polypeptide chain. Proc Natl Acad Sci U S A 47: 1309–14. 13683522 10.1073/pnas.47.9.1309 PMC223141

[2] Kim DE, Blum B, Bradley P, Baker D (2009) Sampling bottlenecks in de novo protein structure prediction. J Mol Biol 393: 249–60. 19646450 10.1016/j.jmb.2009.07.063 PMC2760740

[3] Bowie JU, Eisenberg D (1994) An evolutionary approach to folding small alpha-helical proteins that uses sequence information and an empirical guiding fitness function. Proc Natl Acad Sci U S A 91: 4436–40. 8183927 10.1073/pnas.91.10.4436 PMC43800

[4] Simons KT, Kooperberg C, Huang E, Baker D (1997) Assembly of protein tertiary structures from fragments with similar local sequences using simulated annealing and Bayesian scoring functions. J Mol Biol 268: 209–25. 9149153 10.1006/jmbi.1997.0959

[5] Simons KT, Bonneau R, Ruczinski I, Baker D (1999) Ab initio protein structure prediction of CASP III targets using ROSETTA. Proteins Suppl 3: 171–6. 10.1002/(sici)1097-0134(1999)37:3+<171::aid-prot21>3.3.co;2-q 10526365

[6] Rohl CA, Strauss CEM, Misura KMS, Baker D (2004) Protein structure prediction using Rosetta. Methods Enzymol 383: 66–93. 15063647 10.1016/S0076-6879(04)83004-0

[7] Leaver-Fay A, Tyka M, Lewis SM, Lange OF, Thompson J, et al (2011) ROSETTA3: an objectoriented software suite for the simulation and design of macromolecules. Methods Enzymol 487: 545–74. 21187238 10.1016/B978-0-12-381270-4.00019-6 PMC4083816

[8] Kinch L, Yong Shi S, Cong Q, Cheng H, Liao Y, et al (2011) CASP9 assessment of free modeling target predictions. Proteins: Structure, Function, and Bioinformatics 79: 59–73. 10.1002/prot.23181 PMC3226891 21997521

[9] Xu D, Zhang Y (2012) Ab initio protein structure assembly using continuous structure fragments and optimized knowledge-based force field. Proteins 80: 1715–1735. 22411565 10.1002/prot.24065 PMC3370074

[10] Lee J, Kim SY, Joo K, Kim I, Lee J (2004) Prediction of protein tertiary structure using PROFESY, a novel method based on fragment assembly and conformational space annealing. Proteins 56: 704–14. 15281124 10.1002/prot.20150

[11] Chikenji G, Fujitsuka Y, Takada S (2003) A reversible fragment assembly method for de novo protein structure prediction. The Journal of Chemical Physics 119: 6895–6903.

[12] Karplus K, Karchin R, Draper J, Casper J, Mandel-Gutfreund Y, et al (2003) Combining localstructure, fold-recognition, and new fold methods for protein structure prediction. Proteins 53 Suppl 6: 491–6. 14579338 10.1002/prot.10540

[13] Jones DT, McGuffin LJ (2003) Assembling novel protein folds from super-secondary structural fragments. Proteins 53 Suppl 6: 480–5. 14579336 10.1002/prot.10542

[14] Hamelryck T, Kent JT, Krogh A (2006) Sampling realistic protein conformations using local structural bias. PLoS Comput Biol 2: e131. 17002495 10.1371/journal.pcbi.0020131 PMC1570370

[15] Li SC, Bu D, Xu J, Li M (2008) Fragment-HMM: a new approach to protein structure prediction. Protein Sci 17: 1925–34. 18723665 10.1110/ps.036442.108 PMC2578800

[16] DeBartolo J, Colubri A, Jha AK, Fitzgerald JE, Freed KF, et al (2009) Mimicking the folding pathway to improve homology-free protein structure prediction. Proc Natl Acad Sci U S A 106: 3734–3739. 19237560 10.1073/pnas.0811363106 PMC2656149

[17] Adhikari AN, Freed KF, Sosnick TR (2012) De novo prediction of protein folding pathways and structure using the principle of sequential stabilization. Proc Natl Acad Sci U S A 109: 17442–17447. 23045636 10.1073/pnas.1209000109 PMC3491489

[18] Simoncini D, Berenger F, Shrestha R, Zhang KYJ (2012) A probabilistic fragment-based protein structure prediction algorithm. PLoS One 7: e38799. 22829868 10.1371/journal.pone.0038799 PMC3400640

[19] Blow DM, Rossmann MG (1961) The single isomorphous replacement method. Acta Crystallographica 14: 1195–1202.

[20] Gront D, Kulp DW, Vernon RM, Strauss CEM, Baker D (2011) Generalized fragment picking in Rosetta: design, protocols and applications. PLoS One 6: e23294. 21887241 10.1371/journal.pone.0023294 PMC3160850

[21] Kirkpatrick S, Gelatt CD, Vecchi MP (1983) Optimization by simulated annealing. Science 220: 671–680. 17813860 10.1126/science.220.4598.671

[22] Lourenco H, Martin O, Stutzle T (2001) Iterated Local Search. In “Handbook of Metaheuristics”, Ed F Glover and G Kochenberger, ISORMS 57, p 321–353 (2002), Kluwer.

[23] Berenger F, Zhou Y, Shrestha R, Zhang KYJ (2011) Entropy-accelerated exact clustering of protein decoys. Bioinformatics 27: 939–45. 21310747 10.1093/bioinformatics/btr072

[24] Winn MD, Ballard CC, Cowtan KD, Dodson EJ, Emsley P, et al (2011) Overview of the CCP4 suite and current developments. Acta Crystallogr D Biol Crystallogr 67: 235–42. 21460441 10.1107/S0907444910045749 PMC3069738

[25] Kabsch W (1976) A solution of the best rotation to relate two sets of vectors. Acta Crystallographica A32: 922–923.

