# Supplementary figures and images for: Efficient Sampling in Fragment-Based Protein Structure Prediction Using an Estimation of Distribution Algorithm
Source: PLoS One. 2013 Jul 25;8(7):e68954. doi: 10.1371/journal.pone.0068954 (PMC3723781; doi:10.1371/journal.pone.0068954)

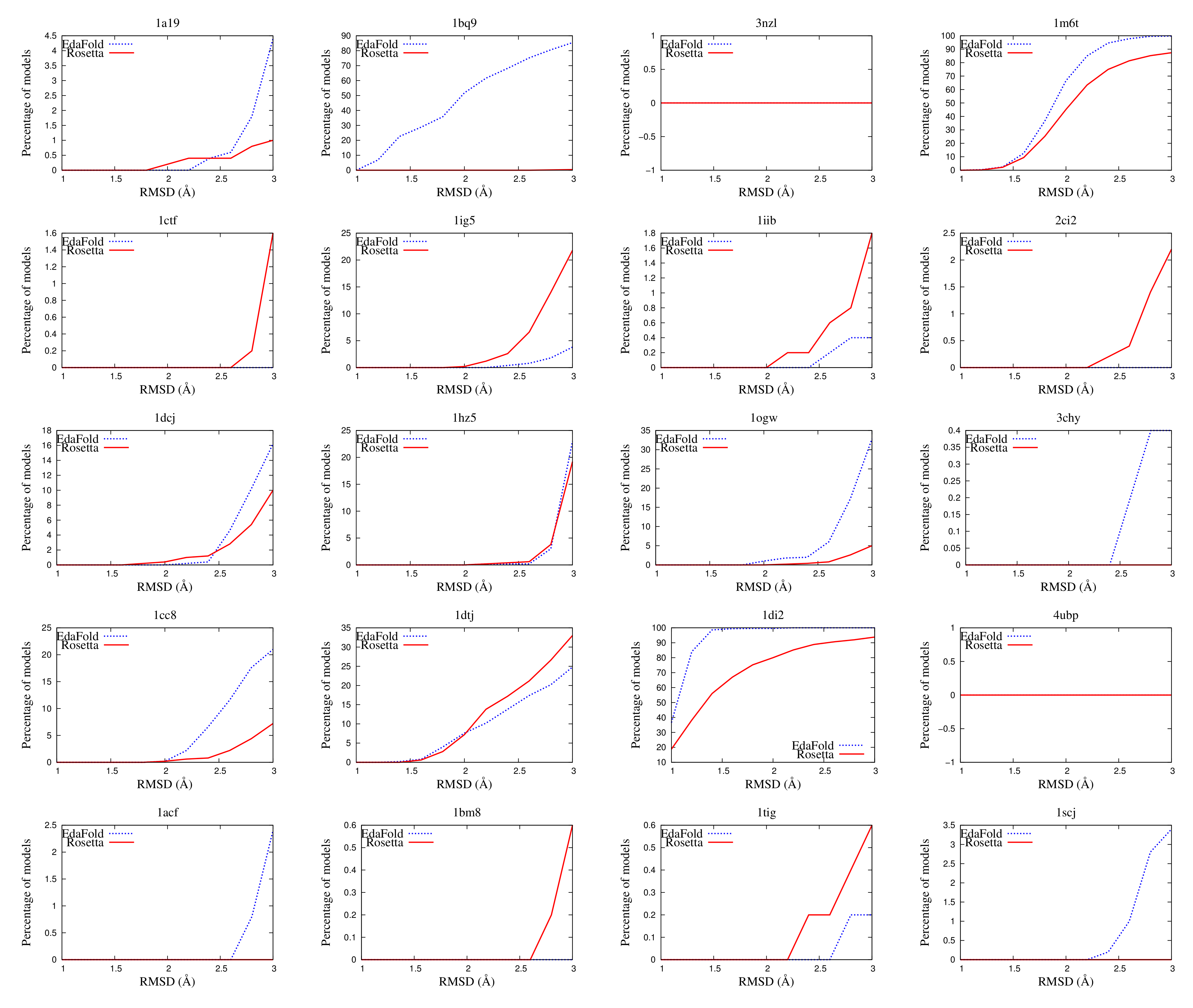

Supplement: Figure S1 — Models distribution as a function of C α RMSD to native for the lowest 500 energies in and datasets. (TIF) [file pone.0068954.s001.tif]
