# Supplementary material for: Efficient Sampling in Fragment-Based Protein Structure Prediction Using an Estimation of Distribution Algorithm
Source: PLoS One. 2013 Jul 25;8(7):e68954. doi: 10.1371/journal.pone.0068954 (PMC3723781; doi:10.1371/journal.pone.0068954)
Supplement: Table S1 — Comparison of all-atom models generated by and . 1‰ AARMSD is the average over the 1‰ lowest AARMSD to native models. Similarly, 1% AARMSD is the average over the 1% lowest AARMSD to native models. Best model is the single lowest AARMSD to native model. All mean differences are statistically significant with a confidence greater than 95% according to the Student's t-test. (PDF) [file pone.0068954.s002.pdf]

Efficient sampling in fragment-based protein structure prediction using an estimation of distribution algorithm

David Simoncini, Kam Y. J. Zhang*
Zhang Initiative Research Unit, Institute Laboratories, RIKEN, Wako, Saitama, Japan
* E-mail: kamzhang@riken.jp

Supporting information: Table S1

Table S1. Comparison of all-atom models generated by *EdaFold*$_{AA}$ and *Rosetta*. 1 ‰AARMSD is the average over the 1 ‰lowest AARMSD to native models. Similarly, 1 % AARMSD is the average over the 1 % lowest AARMSD to native models. Best model is the single lowest AARMSD to native model. All mean differences are statistically significant with a confidence greater than 95% according to the Student's t-test.

	1 ‰AARMSD (Å)		1 % AARMSD (Å)		Best model (Å)	
Target	$EdaFold_{AA}$	*Rosetta*	$EdaFold_{AA}$	*Rosetta*	$EdaFold_{AA}$	*Rosetta*
1bq9	1.39	3.79	2.06	5.04	0.96	2.77
1di2	1.53	1.71	1.70	2.19	1.32	1.33
1scj	3.96	4.45	4.81	5.09	3.25	3.46
1hz5	3.20	3.40	3.68	3.77	1.97	2.61
1cc8	2.75	3.22	3.08	3.87	2.35	2.67
1ctf	3.90	3.54	5.02	4.31	3.08	2.80
1ig5	2.86	2.90	3.26	3.33	2.26	2.18
1dtj	3.04	3.00	4.12	4.06	1.95	2.11
1ogw	2.69	3.21	3.21	3.68	1.93	2.34
1dcj	3.28	3.41	3.74	4.19	2.67	2.39
2ci2	4.11	3.83	5.71	4.99	3.00	3.01
3nzl	4.64	4.76	5.13	5.42	4.00	3.93
1a19	3.82	4.18	4.45	5.18	3.22	2.99
1tig	4.04	4.00	4.47	4.66	2.54	3.10
1bm8	4.54	4.39	5.58	5.35	3.71	3.31
4ubp	4.77	4.51	5.52	5.34	3.55	3.13
1m6t	1.88	2.12	2.14	2.51	1.65	1.80
1iib	3.30	3.45	4.34	4.69	2.35	2.45
1acf	3.71	5.08	4.44	6.40	2.79	3.31
3chy	4.08	4.15	5.20	5.11	2.94	3.21
Average	3.38	3.66	4.08	4.46	2.57	2.74
