# Supplementary material for: Efficient Sampling in Fragment-Based Protein Structure Prediction Using an Estimation of Distribution Algorithm
Source: PLoS One. 2013 Jul 25;8(7):e68954. doi: 10.1371/journal.pone.0068954 (PMC3723781; doi:10.1371/journal.pone.0068954)
Supplement: Table S2 — Comparison of best all-atom models selected based on energy. The first prediction is the model with the lowest energy. The best prediction is the best model out of the five lowest energies. All results are shown as AARMSD to native structure. (PDF) [file pone.0068954.s003.pdf]

Efficient sampling in fragment-based protein structure prediction using an estimation of distribution algorithm

David Simoncini, Kam Y. J. Zhang*
Zhang Initiative Research Unit, Institute Laboratories, RIKEN, Wako, Saitama, Japan
* E-mail: kamzhang@riken.jp

Supporting information: Table S2

Table S2. Comparison of best all-atom models selected based on energy. The first prediction is the model with the lowest energy. The best prediction is the best model out of the five lowest energies. All results are shown as AARMSD to native structure.

	First prediction (Å)		Best prediction (Å)	
Target	$EdaFold_{AA}$	*Rosetta*	$EdaFold_{AA}$	*Rosetta*
1bq9	1.49	4.60	1.52	4.60
1di2	1.86	1.90	1.64	1.76
1scj	8.21	7.75	4.15	6.85
1hz5	3.87	3.95	3.78	3.82
1cc8	4.40	8.61	4.15	3.75
1ctf	7.78	5.28	4.96	3.33
1ig5	7.05	3.24	4.19	3.24
1dtj	2.47	2.40	2.49	2.40
1ogw	3.07	3.28	3.07	3.28
1dcj	5.55	3.53	3.33	3.24
2ci2	8.31	9.08	7.46	7.09
3nzl	6.79	6.37	6.79	6.06
1a19	3.73	4.81	3.73	3.85
1tig	4.84	4.58	4.43	4.49
1bm8	9.70	4.30	4.10	4.30
4ubp	11.19	10.99	6.34	9.34
1m6t	2.71	2.56	2.06	2.35
1iib	2.71	15.16	2.71	10.13
1acf	4.00	3.31	3.66	3.31
3chy	4.94	12.80	4.94	5.74
Average	5.23	5.93	3.98	4.65
