# Supplementary material for: Efficient Sampling in Fragment-Based Protein Structure Prediction Using an Estimation of Distribution Algorithm
Source: PLoS One. 2013 Jul 25;8(7):e68954. doi: 10.1371/journal.pone.0068954 (PMC3723781; doi:10.1371/journal.pone.0068954)
Supplement: Table S3 — Blind selection ability of all-atom models generated by at iterations and . The first prediction is the model with the lowest energy. The best prediction is the best model out of the five lowest energies. All results are shown as AARMSD to native structure. Models produced at iteration alone and iteration alone are compared. For comparison, the columns show the same data obtained from a sample of models randomly picked from 's prediction results. (PDF) [file pone.0068954.s004.pdf]

Efficient sampling in fragment-based protein structure prediction using an estimation of distribution algorithm

David Simoncini, Kam Y. J. Zhang*
Zhang Initiative Research Unit, Institute Laboratories, RIKEN, Wako, Saitama, Japan
* E-mail: kamzhang@riken.jp

Supporting information: Table S3

Table S3. Blind selection ability of all-atom models generated by $EdaFold_{AA}$ at iterations 1 and 4. The first prediction is the model with the lowest energy. The best prediction is the best model out of the five lowest energies. All results are shown as AARMSD to native structure. Models produced at iteration 1 alone and iteration 4 alone are compared. For comparison, the columns *Rosetta* show the same data obtained from a sample of *Rosetta* models randomly picked from *Rosetta*'s prediction results.

	First prediction (Å)			Best prediction (Å)		
Target	Iter. 1	Iter. 4	*Rosetta*	Iter. 1	Iter. 4	*Rosetta*
1bq9	6.88	1.52	9.56	3.38	1.24	6.66
1di2	1.92	1.68	1.92	1.72	1.64	1.68
1scj	4.15	8.38	7.75	4.15	3.64	6.85
1hz5	4.11	3.95	4.59	3.72	3.71	3.96
1cc8	4.26	4.07	8.61	3.89	3.65	3.39
1ctf	6.31	7.78	5.28	5.30	6.64	3.75
1ig5	3.67	7.05	3.41	3.20	6.95	3.20
1dtj	3.33	2.39	2.40	2.32	2.37	2.40
1ogw	3.54	3.37	3.28	3.54	3.09	3.28
1dcj	3.51	5.55	4.76	3.51	3.29	3.25
2ci2	8.17	8.31	7.89	7.78	7.46	7.89
3nzl	11.50	12.21	6.37	6.49	6.46	6.00
1a19	7.61	4.61	4.81	4.13	3.72	3.80
1tig	5.06	4.43	4.88	4.62	4.43	4.88
1bm8	9.73	9.70	4.78	9.73	4.10	3.80
4ubp	10.03	12.48	10.99	6.38	8.33	8.46
1m6t	2.80	2.52	2.35	2.08	2.06	2.10
1iib	10.40	2.71	15.98	10.27	2.71	7.54
1acf	11.07	4.00	8.81	9.22	3.60	5.83
3chy	14.15	4.94	9.72	8.46	4.94	6.43
Average	6.61	5.58	6.40	5.19	4.20	4.75
